# Multiparametric MR for non‐invasive evaluation of tumour tissue histological characteristics after radionuclide therapy

**DOI:** 10.1002/nbm.4060

**Published:** 2019-01-28

**Authors:** Mikael Montelius, Oscar Jalnefjord, Johan Spetz, Ola Nilsson, Eva Forssell‐Aronsson, Maria Ljungberg

**Affiliations:** ^1^ Institute of Clinical Sciences, Sahlgrenska Cancer Center, Sahlgrenska Academy, Department of Radiation Physics University of Gothenburg Gothenburg Sweden; ^2^ Department of Medical Physics and Biomedical Engineering Sahlgrenska University Hospital Gothenburg Sweden; ^3^ Institute of Biomedicine, Sahlgrenska Cancer Center, Sahlgrenska Academy, Department of Pathology University of Gothenburg Gothenburg Sweden

**Keywords:** cancer, functional MRI, histopathology, IVIM, tumour therapy response

## Abstract

Early non‐invasive tumour therapy response assessment requires methods sensitive to biological and physiological tumour characteristics.

The aim of this study was to find and evaluate magnetic resonance imaging (MRI) derived tumour tissue parameters that correlate with histological parameters and that reflect effects of radionuclide therapy.

Mice bearing a subcutaneous human small‐intestine neuroendocrine tumour were *i.v*. injected with ^177^Lu‐octreotate. MRI was performed (7 T Bruker Biospec) on different post‐therapy intervals (1 and 13 days) using T2‐weighted imaging, mapping of T2* and T1 relaxation time constants, as well as diffusion and dynamic contrast enhancement (DCE‐MRI) techniques. After MRI, animals were killed and tumours excised. Four differently stained histological sections of the most central imaged tumour plane were digitized, and segmentation techniques were used to produce maps reflecting fibrotic and vascular density, apoptosis, and proliferation. Histological maps were aligned with MRI‐derived parametric maps using landmark‐based registration. Correlations and predictive power were evaluated using linear mixed‐effects models and cross‐validation, respectively.

Several MR parameters showed statistically significant correlations with histological parameters. In particular, three DCE‐MRI‐derived parameters reflecting capillary function additionally showed high predictive power regarding apoptosis (2/3) and proliferation (1/3). T1 could be used to predict vascular density, and perfusion fraction derived from diffusion MRI could predict fibrotic density, although with lower predictive power.

This work demonstrates the potential to use multiparametric MRI to retrieve important information on the tumour microenvironment after radiotherapy. The non‐invasiveness of the method also allows longitudinal tumour tissue characterization. Further investigation is warranted to evaluate the parameters highlighted in this study longitudinally, in larger studies, and with additional histological methods.

**Abbreviations used**
ADC: apparent diffusion coefficient
AT: arrival time
BE: brevity of enhancement
D: diffusion coefficient
D*: Pseudo diffusion coefficient
DCE: dynamic contrast‐enhanced
DWI: diffusion weighted MRI
f: perfusion fraction
FD: Fibrotic density
IFP: Interstitial fluid pressure
IS: initial slope
IVIM: intravoxel incoherent motion
MVD: microvascular density
ROI: region‐of‐interest
SEmax: relative maximum signal enhancement
T1, T2, T2*: magnetic relaxation time constants
TIC: MR signal time‐intensity curve
TOP: time of peak intensity
TTP: time to peak
WI: wash in
WO: wash out

## 1 INTRODUCTION

Neuroendocrine tumours (NETs) are slow‐growing malignancies and metastases are often found at the time of diagnosis. Currently available treatment options have limited effect on patient survival. New therapeutic options are being developed, such as radionuclide therapy (RNT) using the radiolabelled somatostatin analogue octreotate (^177^Lu‐octreotate). This compound binds to receptors expressed on tumour cells. Thereby the radiation dose to primary and metastatic tumours is maximized, while exposure of healthy tissue to radiation is limited. ^177^Lu‐octreotate shows great potential for treatment of NETs, but optimization of therapy protocols is required. To facilitate this, non‐invasive methods for prediction and assessment of therapeutic effects are needed.

Tumour size is conventionally regarded as a response endpoint. It can be accurately determined using computed tomography (CT) or magnetic resonance (MR) imaging, but is becoming obsolete for evaluating modern, targeted therapies. Methods that are sensitive to the targeted biological processes, such as angiogenesis, proliferation and apoptosis are required instead.^1^, ^2^, ^3^

Histological methods provide unambiguous characterization of biological features of tumours, but examining an entire tumour precludes longitudinal assessment, while biopsies only provide information on the focally sampled tissue regions. Erroneous sampling and problematic anatomical locations of tumours are further limiting factors.

Several functional MR imaging methods are currently being investigated for their ability to indirectly measure tumour characteristics. Promising results regarding early assessment of tumour response to RNT have been found.^1^, ^4^ For example, dynamic contrast‐enhanced MR imaging (DCE‐MRI) is used in oncological imaging to reveal abnormal biokinetic interactions between tumour tissue and contrast material. Semi‐quantitative or quantitative parametric analysis or modelling of the DCE‐MRI signal‐time curve can provide important information on, *e.g*., spatial distribution of vascularity, vessel structure and function, oxygenation, perfusion, and blood volume.^5^ Diffusion weighted MRI (DWI) is commonly used to measure structural restrictions on the Brownian motion of tissue water molecules. Measures obtained can exclude (diffusion coefficient, D) or include (apparent diffusion coefficient, ADC) perfusion related water molecule motion. In addition, DWI can be used to determine the fractional tissue volume of actively perfused microvasculature (*f*).^6^ Quantification of magnetic relaxation times (*T1*, *T2,* and T2*) can reveal altered molecular organization, presence of macromolecules after microvasculature restructuring or leakage, or changes in tissue deoxyhaemoglobin levels.^7^, ^8^

Furthermore, the interest in combining several MR techniques in a multiparametric MR (mpMR) approach is increasing, since it would enable a non‐invasive, spatiotemporally resolved, comprehensive evaluation of several dynamic and functional biological mechanisms that are associated with tumour response.^1^, ^9^, ^10^, ^11^, ^12^

In previous studies, individual MR‐derived tissue parameters for tumour response assessment (*e.g*. water diffusion, vascular parameters, perfusion and hypoxia) have been correlated with biological measures. However, the results sometimes show weak, diverging or even contradictory results, most likely due to the diversity of tumour types, therapeutic modalities, experimental conditions and methodologies described in the literature.^13^, ^14^ Hence, developing a reliable and sensitive response assessment method based on mpMR necessitates a comprehensive evaluation of the link between the pathophysiological processes and the MR‐derived tissue parameters for each combination of tumour type and treatment modality studied.

For MR‐derived parameters to provide robust *in vivo* tumour therapy response assessment, the MR‐methods must be better optimized and understood, both regarding acquisition and evaluation of data, and the parameters must be evaluated with corresponding histological tissue data (*e.g. Dominietto & Rudin, 2013* 11). To our knowledge, the biological interpretation of mpMR data of NETs receiving RNT has not been studied using histological methods.

## 2 AIM

The aim of this project was to evaluate the potential of mpMR for response assessment of NETs receiving RNT by studying correlations between *in vivo*, MR‐derived tissue parameters and biological characteristics of corresponding *ex vivo* tissue samples.

## 3 MATERIALS & METHODS

### 3.1 Tumour model and therapeutics

Samples from the human small‐intestine neuroendocrine tumour model (GOT1^15^) were xenografted subcutaneously on female BALB/c nude mice (*n* = 21, Charles River, Wilmington, MA, USA) that were fed with a standard diet and water *ad libitum*. Each animal grew one tumour in the neck region, which will be referred to as tumour 1 for animal 1, tumour 2 for animal 2, etc. As tumours reached 10–20 mm in diameter, 15 MBq ^177^Lu‐octreotate (specific activity: 26 MBq/μg octreotate, IDB Holland, Baarle‐Nassau, the Netherlands) was injected intravenously, resulting in an absorbed dose to the tumour of approximately 4 Gy (cf. Dalmo *et al*., 2017^16^). The initial 21 animals were included in our mpMR project, including studies on the longitudinal behaviour of MR‐derived tissue parameters in response to therapy^4^ and optimization of image post processing techniques,^17^ both with different specific aims and results. Based on inclusion criteria described below, *n* = 5 of the 21 animals were evaluated in the current study.

The Gothenburg Ethical Committee on Animal Research approved this study.

### 3.2 Overall experimental setup

To correlate MRI data with a broad range of biological response effects, rather than with effects from therapeutic doses, and thereby to increase the biological variability of the investigated parameters, we used a non‐curative amount of ^177^Lu‐octreotate (15 MBq) and also included data from different time points after treatment.

Imaging experiments including the methods mentioned in Table 1 were conducted on days −1, 1, 3, 8, and 13 after treatment (day 0), whereof data from days 1, 8 and 13 are included in the current study. The T2 weighted images were used to verify partial tumour response (reduced tumour volume or reduced growth rate during the first week after treatment, followed by stable or increasing tumour volume during the second week, data not shown) as previously described.^18^

**Table 1:** MR examinations and pulse sequence parameters

MR technique & pulse sequence	Pulse sequence parameters
IVIM‐DWI	3 orthogonal gradient directions, gradient separation/duration: 9/4 ms
	12 *b*‐values: 0, 5, 10, 20, 35, 50, 75, 100, 200, 400, 600 and 800 s/mm^2^
2D SE‐EPI	TR: 1500 ms, TE: 21 ms, number of averages/segments: 3/1
	Effective bandwidth ≈ 300 kHz
	Partial Fourier acceleration: 1.5
	Pixel size: 320^2^ μm^2^, slice thickness: 1000 μm, slice gap: 500 μm
	Fat suppression: Frequency selective
	Scan time < 6 minutes
T2*‐mapping	10 echoes (TE): 5, 10, 15, … 50 ms
	TR: 2000 ms, number of averages: 1, flip angle: 30°
Multiple echo gradient echo (MGE)	Slice positions imported from IVIM‐DWI experiment
	Pixel size: 160^2^ μm^2^, slice thickness: 1000 μm
	No fat suppression scan time < 4 minutes
T1‐mapping	7 TR: 13000, 9000, 4500, 2500, 1500, 750, 300 ms
	TE: 24 ms, number of averages: 1, RARE factor: 4
2D RARE (RAREVTR)	Refocusing flip angle: 180°
	Pixel size: 280^2^ μm^2^, slice thickness: 1000 μm, single slice
	Slice positions imported from IVIM‐DWI experiment (central slice)
	No fat suppression
	Scan time < 10 minutes
DCE‐MRI	Number of repetitions (dynamics): 100, temporal resolution: 4.2 s (varied slightly with FOV), contrast injection during sixth dynamic
2D RARE	TR: 300 ms, TE: 24 ms, number of averages: 1, RARE factor: 4
	Partial Fourier acceleration: 1.5
	Pixel size: 280^2^ μm^2^, slice thickness: 1000 μm, single slice
	Slice positions imported from IVIM‐DWI experiment (central slice)
	Fat suppression: Frequency selective
	Scan time < 8 minutes
T2 weighted MRI	TR: 4190 ms, TE: 45 ms, number of averages: 2, RARE factor: 6
	Pixel size: (160 ± 50)^2^ μm^2^ (varied with FOV)
2D RARE	Slice thickness: 700 μm, no slice gap
	Fat suppression: Frequency selective
	Scan time < 4 min

The 5/21 tumours included in this study fulfilled the following requirements: 1) they were successfully imaged in the MR experiment preceding tumour extraction, 2) they were adequately located, shaped and sized for the resection method used, 3) they were successfully stained at histology, and 4) anatomical landmarks were recognizable on both histological sections and MR images. These inclusion criteria resulted in 4/5 included tumours (no. 1, 3, 4 and 5) being harvested on day 13 and 1/5 included tumours (no. 2) being harvested on day 1.

During imaging, animals were anaesthetized using air and isoflurane (2–3%, MSD Animal Health, Copenhagen, Denmark), body temperature was maintained using a circulating warm water system and a heating pad, and a pressure sensitive pad was used to monitor respiration (SA Instruments, Inc., NY, USA). For contrast‐material administration during image acquisition, a peripheral venous catheter was fixed to the tail vein and connected to a 1 ml syringe outside the magnet bore via a 70 cm infusion line.

Immediately after the MRI experiment preceding the tumour extraction, animals received a lethal intraperitoneal injection of sodium pentobarbitone (Pentobarbitalnatrium vet, Apotek Produktion & Laboratorier AB, Huddinge, Sweden, 60 mg/ml) followed by heart incision, tumour extraction and histological preparation.

### 3.3 MRI experiments

MRI examinations were performed using a 20‐cm inner‐diameter horizontal bore 7 T MR system with 400 mT/m gradients (Bruker BioSpin MRI GmbH, Ettlingen, Germany; software: ParaVision 5.1).

A 4‐channel array rat brain receiver coil (RAPID Biomedical GmbH, Rimpar, Germany) and a 72‐mm volume coil were used to image tumours 1 and 3, 4, and 5, and a 50‐mm quadrature transmit/receive volume coil was used to image tumour 2 (RAPID Biomedical GmbH, Germany).

Field‐map based shimming (MAPSHIM) was used to improve the field homogeneity within the tumour. Transversal images of the longitudinal position of the animal with greatest tumour area were acquired using a field of view (FOV) that covered the tumour extent, including the following methods: Multi b‐value diffusion‐weighted imaging for intravoxel incoherent motion modelling (IVIM‐DWI), T2* and *T1* quantification, dynamic contrast‐enhanced MRI (DCE‐MRI: 0.1 M Gd‐DTPA, DOTAREM, Gothia Medical, Billdal, Sweden, 0.3 mmol/kg bodyweight, tail *i.v*. injection during the 6^th^ dynamic) and T2‐weighted MRI.

Imaging experiments were performed on the same day the animal was killed for tumour tissue harvesting, except for the DCE‐MRI experiments on animals 1, 3, 4, and 5, which were conducted on day 8 instead of day 13 for logistical reasons involving the other studies. Total scan time was approximately 90 min. Detailed acquisition parameters are given in Table 1.

### 3.4 MR image post processing

Post processing was performed on a voxel‐by‐voxel basis using MATLAB (R2015b, The MathWorks, Inc., Natick, MA, USA) with standard and in‐house developed functions, scripts and graphical user interfaces. A 2 × 2 median filter was applied to all images for noise reduction before further post‐processing. The 19 parameters derived from MRI examinations are defined in Table 2.

**Table 2:** MR parameter definitions

Parameter		Description
**Diffusion weighted imaging (DWI)**		
		Intravoxel incoherent motion (IVIM) $S\left(b\right)=S_{0}\left(1-f\right)\cdot e^{-\mathrm{bD}}+S_{0}f\cdot e^{-b\left(D+D^{*}\right)}$ *S*_*0*_: Signal without diffusion weighting A voxelwise Bayesian method with uniform prior distributions and mode as central tendency measure was used to estimate the model parameters^17^ Parameter limits were *D*: [0, 5] μm^2^/ms, *f*: [0, 1], D*: [0, 1000] μm^2^/ms, *S*_*0*_: [0, 2 × *S*_*max*_], where *S*_*max*_ is the maximum measured signal
*D*	Diffusion coefficient	The tissue water diffusion coefficient
D*	Pseudo diffusion coefficient	Perfusion related pseudo diffusion coefficient of incoherently flowing blood in the tissue
*f*	Perfusion fraction	Signal fraction of from incoherently flowing blood in the tissue
		Apparent diffusion *S*(*b*) = *S*_0_*e*^−*b* · *ADC*^ *S*_*0*_: Signal without diffusion weighting *ADC* was estimated from a least squares fit with two b‐values (0 and 800 s/mm^2.^)
*ADC*	Apparent diffusion coefficient	The apparent diffusion coefficient, which includes effects from both diffusion and perfusion
**Relaxation time mapping**		
		T2* relaxation $S\left(\mathrm{TE}\right)=A+Ce^{\frac{-\mathrm{TE}}{T2^{*}}}$ *S (TE)* is the signal intensity for echo time TE *A* = signal bias *C* = signal intensity without T2* relaxation and no bias Least squares fitting was used to estimate T2*. Voxels with the goodness‐of‐fit parameter R^2^ < 0.4 were excluded
T2*	T2* time	Transversal tissue relaxation time
		T1 relaxation $S\left(\mathrm{TR}\right)=A+C\left(1-e^{\frac{-\mathrm{TR}}{T1}}\right)$ *S (TR)* = signal intensity for repetition time TR *A* = signal bias *C* = signal intensity after complete T1 relaxation with no bias Least squares fitting was used to estimate *T1*
*T1*	T1 time	Longitudinal tissue relaxation time
**Dynamic contrast enhancement (DCE) MRI**		
		Semi quantitative characteristics *S(t)*, a continuous representation of signal intensity as a function of time, was determined by fitting a smoothing spline (smoothing parameter = 0.01) *S*_*0*_, the baseline signal intensity, was defined as the mean of the pre‐contrast dynamics *σ*_*voxel*_, the voxel noise, was defined as the standard deviation of the residuals of *S(t)* for each voxel *S*(*t*) > *S*_0_ + 5 · *σ*_*voxel*_ defined significant contrast enhancement *σ*_*image*_, the image noise, was defined as the mean of *σ*_*voxel*_ *S*_0_ > 5 · *σ*_*image*_ defined the threshold for inclusion of voxels in the analysis *S*_*max*_ was defined as the maximum value of *S(t)* for each voxel
*AT*	Arrival time	Time after injection required for signal intensity to reach significant enhancement
*TOP*	Time of peak intensity	Time after injection required to reach maximum signal intensity
*TTP*	Time to peak	Time between *AT* and *TOP*
*SEmax*	Relative, maximum signal enhancement	Maximum signal intensity relative to baseline signal intensity: *(S*_*max*_ *– S*_*0*_ *) /S*_*0*_
*SE60*	Relative signal enhancement at 60 s	Signal intensity at 60 s after injection relative to signal baseline intensity: *[S(60 s) – S*_*0*_ *] /S*_*0*_
*SER*	Signal enhancement ratio	Ratio of early (55 s) and late (300 s) relative signal enhancements
*CER*	Contrast enhancement ratio	Ratio of maximum signal intensity and baseline signal intensity: *S*_*max*_ */S*_*0*_ Missing value if no significant enhancement was obtained
*AUCn*	Normalized area under the curve	Area under *S(t)* between 0 and 5 minutes, normalized to *S*_*0*_
*WI*	Wash in	Maximum time derivative of *S(t)* between *AT* and *TOP*
*WO*	Wash out	Maximum negative time derivative of *S(t)* between *TOP* and the last dynamic. Missing value if <5 dynamics left after *TOP*
*BE*	Brevity of enhancement	Time between the time points of *WI* and *WO*
*IS*	Initial slope	Average rate of signal enhancement between *TOP* and contrast injection: *(S*_*max*_ *‐ S*_*0*_ *) /TOP*
*NS*	Negative slope	Average rate of signal decrease between *TOP* and the last dynamic. Missing value if <5 dynamics left after *TOP*

### 3.5 Tissue sampling, histological processing and digitization

**Figure 1:**
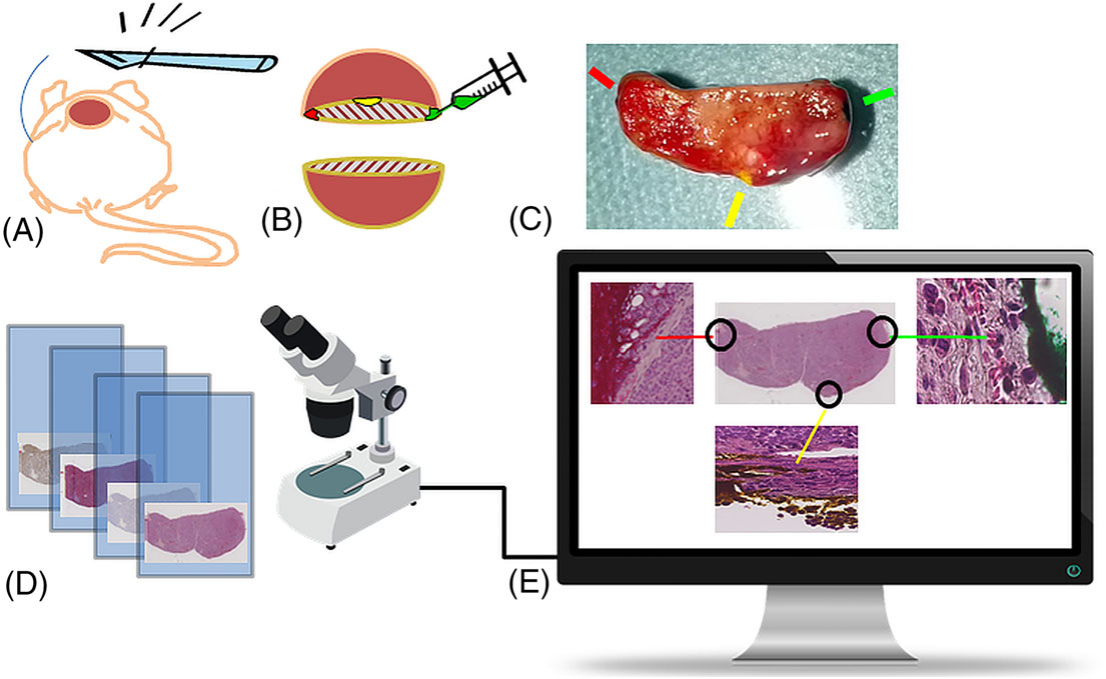
Obtaining digital histology images for alignment with the corresponding MR parameter maps. A) The dead animal is positioned for incision through skin and tumour, with scalpel cutting adjacent and parallel to the imaged tumour plane. B) Colour coding of the imaged tumour plane by tissue ink injections on the left, right and dorsal tumour borders. C) Digital photography of the divided and colour coded tumour, with indications of where the ink is visible. The plane of the surface facing the camera will be parallel to the microtome knife sweep plane. D) Glass microscopy slides with differently stained parallel sections are digitized using a slide scanner. E) The ink, preserved through the preparation and paraffin embedding procedure, is visible on the magnified portions of the digitized HE stained section, and can be used to recover the orientation of the histological sections for alignment with the MR images

Just after the animal had been killed, a scalpel was used to divide the tumour *in situ* by placing a section adjacent and parallel to the imaged tumour plane. The same plane was colour coded with tissue ink on the left, right, and dorsal tumour borders to keep track of anatomical orientation during histological processing. At paraffin embedding (Thermo Scientific™ HM 355S Automatic, Fisher Scientific, Lund, Sweden), the flat tumour surface made by the scalpel was positioned to meet the sweep plane of the microtome knife. When the tissue ink was reached by the microtome, *i.e*. when the imaged tumour plane was reached, serially produced 3‐μm sections were collected for histological and immunohistochemical staining. Digital images of the stained sections were produced using a Leica SCN400 Slide Scanner (40× magnification, Leica Microsystems, Wetzlar, Germany), resulting in images of 0.25 × 0.25 μm^2^ resolution. Image post‐processing was performed in MATLAB, where the tissue ink was located on the magnified images, allowing proper alignment of the digitized histological images and the MR images. Figure 1 visualizes the process of producing digital histology images for subsequent alignment with the corresponding MR parameter maps.

Four stains were applied to adjacent tumour sections: haematoxylin‐eosin (HE, Tissue‐Tek Prisma, Sakura FineTek, Alphen aan den Rijn, the Netherlands), Masson Trichrome (MT, Artisan Link, Dako, Glostrup, Denmark), and antibodies to Ki67 (AB9260; Merck Milipore Burlington, MA, USA) and CD31 (AB28365; Abcam, Cambridge, UK). The latter two were pre‐treated with EnVision™ FLEX Target Retrieval Solution (high pH; PT‐Link; Dako, Glostrup, Denmark) followed by incubations using Envision Flex (Dako, Glostrup, Denmark). The staining was performed in an Autostainer Link (Dako, Glostrup, Denmark) following the manufacturer's instructions. Positive and negative controls were included in each run.

### 3.6 Image registration and data sampling

**Figure 2:**
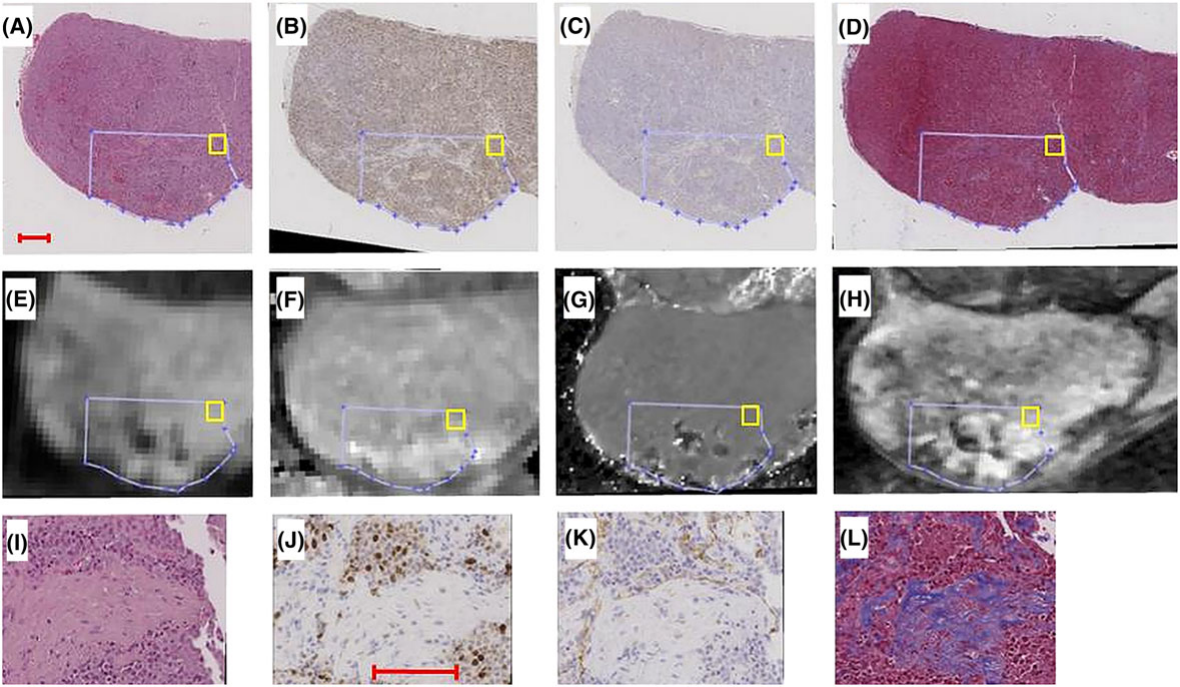
Histological images [(A‐D): HE, Ki67, CD31, MT] and MR images/parametric maps [(E‐H): IVIM‐DWI of b = 600 s/mm^2^, T1 map, T2* map, T2 weighted image] of tumour 2. The sequential image registration and data sampling procedure is as follows: 1) a tumour sub‐region (blue delineation on (A‐H)) is intermutually registered on the histological images, 2) the MR parameter maps of the same region are registered to the T2‐weighted image (H), 3) the final transformation linking all images to each other is established by registering a histological image also to the T2‐weighted image, and 4) a data sampling algorithm systematically extracts data from the entire registered region, before the next tumour sub‐region is manually registered for sampling. The yellow rectangle in (A‐H) shows the position of a first 250 × 250 μm^2^ sample in a registered tumour sub‐region. The average MR parameter values in the sample region are extracted from each parameter map, and the histological indices are calculated for the same position. The next sample is then taken adjacent to the first position, and the procedure is repeated until the entire registered region has been sampled. Enlargements of the sample position shown in (A‐D) are shown in (I‐L). enlargements were used to validate proper registration based on the tissue microscopic landmarks (e.g. region with low cell density on (I‐L)), as well as to reject samples where the magnification revealed histological artefacts, such as cracks or folds. Bars in (A) and (J) indicate 1.0 and 0.1 mm, respectively

Landmark‐based manual image registration using the MATLAB control point selection tool was performed to intermutually align the histological images and to register them to the T2‐weighted MRI. The MR parameter maps were manually registered to the T2‐weighted image by manual adjustment of rotation, translation and scaling. The MATLAB *fitgeotrans* function, which fits a geometric transformation to pairs of landmark points defined in the images to be aligned, was then used to infer the transformation matrices linking all images. Tumour regions affected by obvious histological tears/folds or MR‐related artefacts, or where landmarks could not be identified were avoided by performing the registration and data sampling sequentially on tumour sub‐regions. Figure 2 illustrates how a data sample is taken from a registered tumour sub‐region. Before the automatic sampling process of a registered sub‐region was started, the histological regions corresponding to each planned sample position were sequentially magnified and scrutinized. If an artefact was observed for a particular stain and position, the corresponding histological index was treated as missing data. Similarly, if an MR parameter was not defined according to Table 2 for a particular sample, it was automatically discarded.

### 3.7 Histological indexing

Histological indices were calculated for each sampling position in area units of 250 × 250 μm^2^, *i.e*. dimensions similar to the in‐plane resolution of the MR experiments. The HE segmentation was based on previously presented principles.^19^ In brief, a normalization algorithm was used to account for inconsistencies in the preparation and staining process. Normalized images were converted to grey‐scale intensity images and subjected to a modified watershed segmentation to detect cell shaped objects (round to oval). Colour thresholding was then used to identify tumour cells with dark stained nuclei. Assuming that the dark stain represented condensed chromatin due to apoptosis (*c.f*. Kroemer et al. 2005^20^), the apoptotic tumour cell count (HEcount) was defined as the number of apoptotic cells per area unit.

Ki67 positive tumour cells were identified using the method described for HEcount except normalization, which was unnecessary due to the higher contrast between cells and background tissue. The colour threshold was set to detect only cells with brown‐stained Ki67 positive chromatin. Proliferating tumour cell count (Ki67count) was thus defined as the number of Ki67 positive cells per area unit.

Micro‐vessel density (MVD) and fibrotic density (FD) were determined by colour thresholding only, and defined as the fraction of CD31 and MT positive area per area unit, respectively.

**Figure 3:**
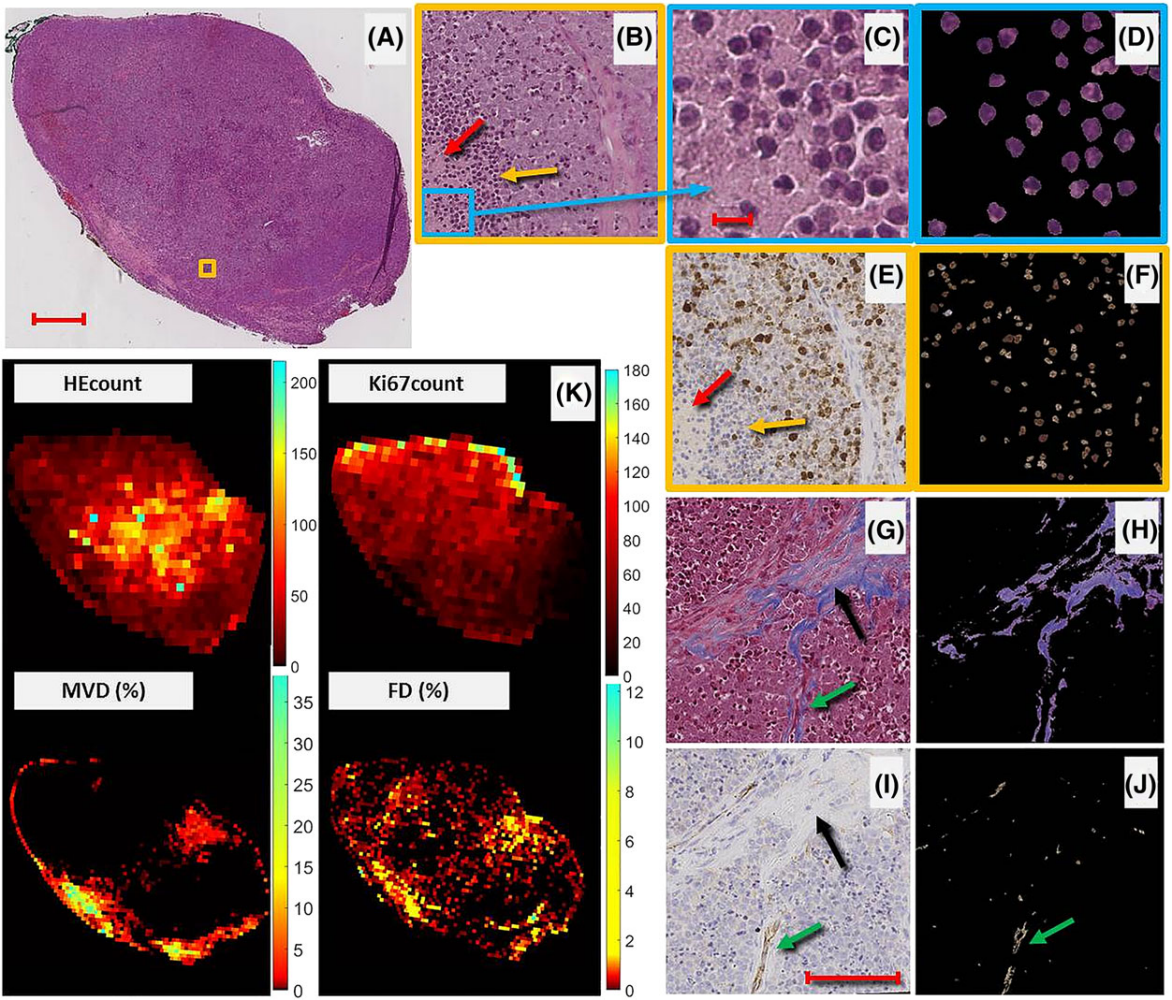
Tissue segmentation and calculation of histological indices. (A) HE stained histological section of tumour 4 with corresponding heat maps (K) of HEcount, Ki67count, MVD and FD. The orange square in (A) is magnified in (B), showing apoptotic regions (densely packed, dark stained nuclei of apoptotic cells, indicated by a yellow arrow), necrotic tissue (red arrow) and viable tumour, as verified by the corresponding Ki67 magnification (brown‐stained cells in (E)). the region in the blue rectangle in (B) is magnified in (C), where haematoxylin positive (dark purple) nuclear fragments of pyknotic (apoptotic) cells are visible. Another region of the same tumour, but stained with MT, is magnified in (G), with a black arrow indicating fibrotic tissue. The corresponding region stained with CD31 (i) reveals formations of blood vessels by the presence of endothelial cells (brown regions indicated with a green arrow). The right panel of the figure (d, f, h, j) shows the results from the segmentation used for index calculations, resulting in indices: HEcount = 395, Ki67count = 136, FD = 12.3% and MVD = 0.7% in b, f, h and j, respectively. Bars in (A, C, I) indicate 1.0, 0.01 and 0.1 mm, respectively

The segmentation and indexing performance was validated by a board‐certified pathologist (ON). Examples are shown in Figure 3.

### 3.8 Data collection & statistics

All data analyses and statistical tests were performed in MATLAB.

Non‐normally distributed MR parameters or histological indices were transformed to approximately normal distributions using the Box‐Cox power transformation (MATLAB *boxcox* function).

A linear mixed‐effects model including intercept and slope for both fixed and random effects was used to investigate the pairwise correlations between each MR parameter and histological index, where tumour number (1‐5) accounted for random effects, such as differences in tissue preparation, staining concentration, colour thresholding, and coil configuration. Observations containing missing values in the pairwise comparisons were omitted. A p‐value <5.0 × 10^−4^ (0.05 adjusted for multiple comparisons) for the fixed effects variable was considered statistically significant.

A statistically significant correlation was further evaluated on a per‐animal basis. Five‐fold cross‐validation of a simple linear regression model between the MR parameter and the histological index was calculated, and the mean squared error of the cross validation, normalized to the squared mean of the histological index, was used to indicate the predictive power of the correlation. The normalization enabled comparison between histological indices.

To study similarities and differences between histological indices and MR parameters, agglomerative hierarchical clustering was performed on the normalized parameters, omitting observations containing missing values. The single linkage distance measure was evaluated due to its invariance to transforms.^21^

## 4 RESULTS

### 4.1 Image quality and registration performance

In general, the 19 MR‐derived tissue parameters that were evaluated (Table 2) produced parametric images of good quality, except for the IVIM pseudo diffusion coefficient D*, which was mostly assigned either of the parameter limit values in the fitting procedure (Table 2). The results based on D* are thus inconclusive.

The image quality of the MR technique most sensitive to image distortions (EPI‐based DWI for estimation of ADC, D, D* and *f*) is shown for tumour 2 in Figure 2. The figure confirms visually that the sub‐regions in the MR images and histological sections were well aligned after image registration.

### 4.2 Tissue segmentation and histological indexing

Results from the automatic segmentation of histological images used for index calculation are shown for tumour 4 in Figure 3. The combination of the algorithm for cell detection and colour thresholding facilitated automatic identification of apoptotic cells in the HE images (Figure 3d) and proliferating cells in the Ki67 images (Figure 3f). Fibrotic tissue (Figure 3h) and vascular endothelial cell presence (Figure 3j) were also adequately identified in the MT and CD31 images, respectively.

Heat maps of the histological indices HEcount, Ki67count, MVD and FD reveal highly heterogeneous and markedly different patterns in the contiguous tumour sections (Figure 3k), which demonstrates a broad range of biological states in the treated tumours.

**Figure 4:**
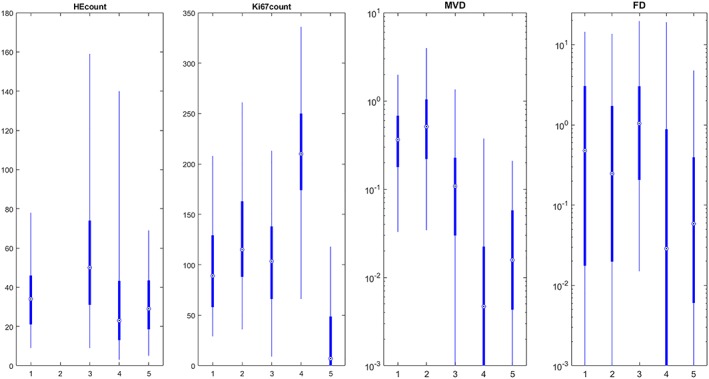
Distribution of histological indices within each tumour (x‐axis) for the four evaluated stains. By visual inspection, the variation of histological indices within tumours appears greater than the variation between tumours, except for, e.g., MVD in tumour 4 and Ki67count in tumour 4 and 5. The indices of tumour 2 (harvested day 1 after therapy) show a distribution similar to the other tumours. Note the logarithmic scale for improved visualization of MVD and FD indices. The number of indices in the HEcount boxplots for tumour 1–5 are *n* = 209, 0, 222, 247, and 117, respectively. The corresponding numbers for Ki67count are: 210, 997, 222, 247 and 128; MVD: 213, 997, 222, 247 and 127; FD: 212, 996, 222, 247 and 125. The dot represents the median value, box edges represent 25^th^ and 75^th^ percentiles and whiskers represent 2.5^th^ and 97.5^th^ percentiles

The distribution of histological indices is shown for each tumour in Figure 4. With some exceptions, there seems to be less variation of indices between tumours than within tumours.

### 4.3 Regression analysis

In total, *n* = 1821 samples could be extracted from the five evaluated tumours (*n* = 215, 998, 228, 252 and 128 samples from tumours 1–5, respectively). The HEcount index was excluded completely from the samples of tumour 2 due to a digitization artefact visible on magnification, and the DCE‐MRI and *T1* parameters were excluded from the tumour 3 samples since no adequate landmarks for registration could be identified. Some MR parameters were excluded from analysis since they were undefined according to Table 2 (*e.g*., if significant contrast enhancement was not reached). A complete overview of the data finally included in the analyses is presented as supplemental material.

**Figure 5:**
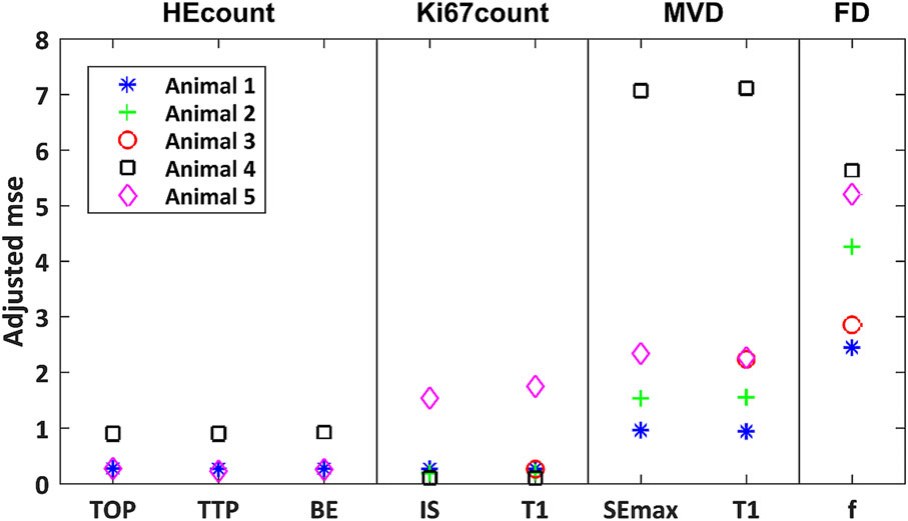
Predictive power of pairwise correlations for individual animals. The predictive power, defined as the mean squared error of 5‐fold cross validation normalized to the squared mean of the histological index, of models constructed from pairwise simple linear regression of MR parameters and histological indices. Data from each investigated tumour (animal) is shown separately

The predictive power of simple linear regression models of the pairwise combinations of MR parameters and histological indices that were significantly correlated on the total sample population level are shown in Figure 5. For the set of evaluated MR parameters, we found a relatively high predictive power for correlations with HEcount and Ki67count, and an intermediate level for MVD. FD seems to be the histological index that is most difficult to evaluate *in vivo* using the MR parameters included in this study.

**Figure 6:**
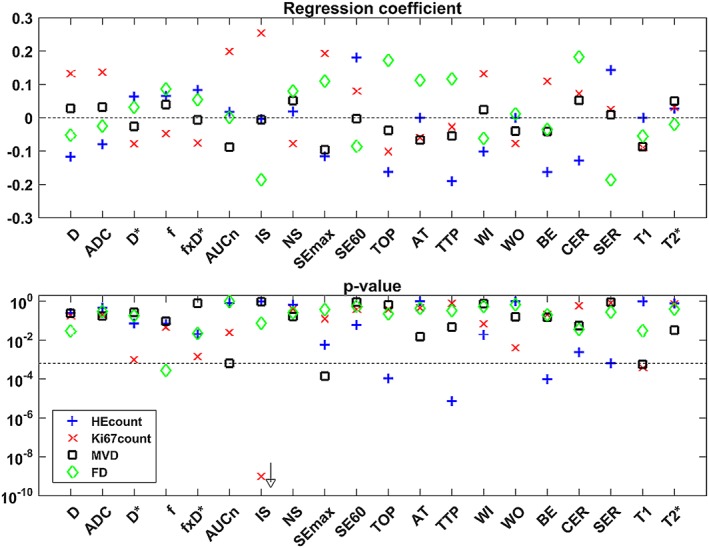
Regression coefficients and p‐values for correlations found by the linear mixed‐effects model. The relation between MR parameters and histological indices, determined for the combined data from all tumour samples (n = 1821) is shown by the regression coefficient (upper plot). The corresponding p‐value is shown in the lower plot, where the vertical dashed line indicates the *p* = 0.05 level after adjustment for multiple comparisons. The p‐value for IS vs. Ki67count was 1.5e‐23. Note that interpretation of D* correlations should be avoided due to poor quality of data

The results from the linear mixed‐effects regression analysis of the total sample population are summarized in Figure 6, and the tumour origin and number of samples from each tumour are shown for each correlation in the supplemental figure. Several combinations of MR parameters and histological indices were correlated on a statistically significant level. Of these, the MR parameters that correlated with HEcount were: time of peak intensity (*TOP*, *n* = 573 samples, regression coefficient (r) = −0.16, p‐value (p) = 1e^−4^); time to peak (*TTP*, *n* = 564, *r* = −0.19, *p* = 7e^−6^); brevity of enhancement (*BE*, *n* = 563, *r* = −0.16, *p* = 1e^−4^). MR parameters correlating with Ki67count were: initial slope (*IS*, *n* = 1355, *r* = 0.25, p = 1e^−9^); T1 time (*T1*, *n* = 1804, *r* = −0.09, *p* = 4e^−4^). MR parameters correlating with MVD were: maximum signal enhancement (*SEmax*, *n* = 1583, r = ‐0.10, p = 1e^−4^); T1 time (*T1*, *n* = 1806, r = −0.09, *p* = 6e^−4^). The MR parameter correlating with FD was the perfusion fraction (*f*, *n* = 1802, *r* = 0.09, *p* = 3e^−4^). The corresponding sample statistics for non‐significant correlations can be found in the supplemental material.

**Figure 7:**
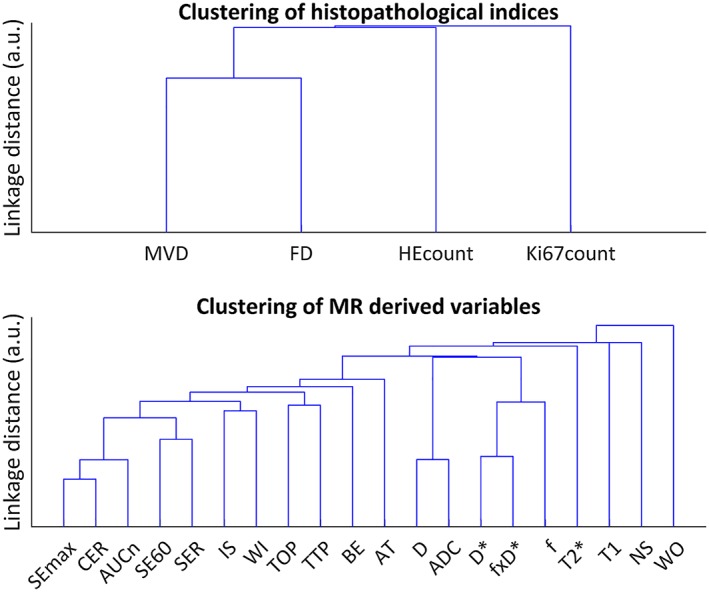
Dendrogram of the cluster analysis of the histological indices (upper), and MR derived parameters (lower)

No obvious clustering was found between the histological indices in the cluster analysis, except for a slightly earlier joining of MVD and FD at approximately 75% of the height of the clade joining all indices (Figure 7, upper dendrogram). The corresponding dendrogram for the MR parameters (Figure 7, lower dendrogram) show that the contrast enhancement ratio (*CER*) and *SEmax,* as well as *D* and *ADC*, contain highly similar information, as expected from similarities in the parameter definitions. Viewed from a perspective of differences (from above in Figure 7), seven overall groups of parameters may be identified: four groups containing single parameter (wash out (*WO*), negative slope (*NS*), *T1* and T2*), one group with the IVIM perfusion parameters (*f*, D* and *f* × D*), one group with the diffusion parameters (*D* and *ADC*) and one group with the remaining DCE‐MRI parameters.

## 5 DISCUSSION

In this work, we demonstrate the potential of multiparametric MRI to non‐invasively characterize tumour tissue treated with ^177^Lu‐octreotate therapy. Previous studies that have evaluated MR‐derived tissue parameters with histology have focussed on one or a few MR parameters, and compared global, averaged tumour MR parameters or histological parameters to address the image registration problems that arise due to deformation of tissue on histological preparation.^9^, ^10^, ^12^ We use a semi‐automatic method to calculate histological indices on a large number of tissue samples, and use a straightforward method based on registration of tumour sub‐regions to accomplish localized correlation analysis.

No standard for spatial registration of histological tissue sections to *in vivo* image data has emerged, but some methods have been proposed.^22^, ^23^ Most of them are in‐house developments with, *e.g*., moulding of subcutaneous tumours or stereotactic assemblies and fiducial markers that preserve spatial orientation throughout the histological preparation. Various image registration techniques, such as rigid or affine and elastic registration have been proposed.^24^ Unfortunately, the laborious nature common to all techniques effectively restricts their use to smaller studies.

We chose to perform the study on mice xenografted with a patient‐derived small‐intestine neuroendocrine tumour (GOT1^15^) treated with ^177^Lu‐octreotate, since we have plenty of experience working with this model. The model tumour retains most of the properties of the patient's tumour. In general, we do not expect the choice of treatment to influence the overall results in the present study. It is, however, likely that RNT and, *e.g*., external radiation therapy would affect the tumour differently due to inhomogeneous vascular supply, resulting in uneven accessibility for RNT.

The primary mechanism of action of radiotherapy is to damage tumour cell DNA. In addition, radiation affects several mechanisms in the tumour microenvironment, including proliferation, vascularization, and tissue degeneration. The results from the linear mixed‐effects regression indicate that it is possible to non‐invasively characterize these effects using mpMR, since all four evaluated histological indices could be predicted by one or several MR parameters (Figure 6).

DCE‐MRI parameters reflecting exchange rate and perfusion (*e.g. TTP*, *TOP* and *BE*) were, to a larger extent than contrast amount‐related parameters (*e.g. AUCn* and *SEmax*), negatively associated with HEcount. These findings match those of other studies^25^, ^26^ (Figure 6). If low *TTP*, *TOP,* and *BE* imply adequate vascular function and perfusion, and thereby delivery of ^177^Lu‐octreotate, increased apoptotic activity in the affected regions can be inferred. It should be noted that TTP, TOP, and BE were measured a week prior to the tumour extraction for HEcount calculation, but it seems plausible that apoptosis was induced in tumour regions that previously were well perfused, and thereby adequately reached by the therapeutic agent. Our study also showed a positive correlation between *IS, i.e*. the initial slope of the TIC, and Ki67count. In tumours, the vascular permeability is often high, and *IS* is therefore considered a measure of perfusion.^27^, ^28^, ^29^ This agrees with our results, since perfused tumour regions should exhibit higher proliferative activity due to adequate supply of nutrients and oxygen. For similar reasons, *IS* may also reflect tissue oxygen status. In a rat model of prostate cancer, rapid rise of the TIC (similar to high *IS*) was found in tumour regions that were less hypoxic on histology and *vice versa*.^30^ Tissue hypoxia is associated with malignant progression, metastatic potential, angiogenesis, and increased interstitial fluid pressure (IFP),^1^, ^29^, ^31^ which makes *IS* a highly interesting, non‐invasive biomarker for tumour response assessment.

The linear models of the functional DCE‐MRI parameters (*TOP, TTP, BE, IS*) predicted the histological indices with relatively high power (Figure 5), which supports the assumption of fairly linear relationships. Furthermore, the cluster analysis (Figure 7) shows that the parameters contain relatively unique information within the largest DCE‐MRI cluster (*AT* and left, Figure 7), underlining the importance of extracting multiparametric information in evaluations of tumour tissue.

Tissue *T1* was negatively correlated with both MVD and Ki67count (Figure 6). *T1* is affected by variations in macromolecular concentration, and increased MVD is often associated with angiogenesis and high microvascular permeability in tumours.^32^, ^33^, ^34^ Plasma proteins leaking into the extracellular extravascular space (EES) and accumulating due to inadequate lymphatic drainage may thus have reduced *T1*. Increased biosynthesis of macromolecules related to proliferation may have had the same effect. This finding is consistent with *T1* increases that have been observed after vascular normalization induced by anti‐angiogenic drugs.^7^, ^31^, ^35^ For example, *T1* increased when microvascular density and proliferation (determined from CD31 and Ki67 staining, respectively) decreased in a mouse model of ovarian adenocarcinoma receiving anti‐angiogenic therapy.^7^

Angiogenesis/MVD and proliferation are clinically relevant tumour characteristics, which makes *T1* highly interesting as a non‐invasive imaging biomarker that should be further studied. Furthermore, *T1* had relatively high predictive power for Ki67count and, unlike *IS*, *T1* would not require contrast injection for Ki67count assessment. Nevertheless, *T1* and *IS* contain complementary information as shown by the cluster analysis (Figure 7). Indeed, *T1* reflects an environmental characteristic rather than a functional perfusion‐related characteristic, and both parameters may be useful in a multiparametric approach for tumour tissue characterization.

Tissue T2* did not correlate with any of the histological parameters in the present study. T2* is sensitive to magnetic field inhomogeneities caused by, *e.g*., deoxyhaemoglobin concentrations, and it has also been associated with necrosis.^22^ It may, however, be problematic to study T2* correlations with parameters acquired after a certain delay (*e.g*. between imaging and subsequent tissue fixation) due to temporal variations in tissue oxygenation, a phenomenon often observed in solid tumours.^36^ It is also possible that CD31‐based MVD, the parameter we anticipated would correlate with T2*, is a poor choice for assessing regional perfusion properties, and thereby oxygen status in tumours.

No correlations were found between diffusion parameters (*D* and *ADC*) and the evaluated histological indices in this study. Diffusion is affected by the size and viscosity of the EES, membrane structures in cellular debris, and other factors. Although viscosity may not be reflected by any of the evaluated indices, it seems plausible that the level of apoptosis, proliferative activity, or fibrosis should be reflected in Ki67count, HEcount, or FD. Due to the lack of landmarks for registration in central parts of the tumours, however, a majority of the data is based on more peripheral parts of the tumours (typically outside half the tumour radius). Reduced variation of the evaluated parameters may thus have obscured correlations. More likely, however, only day 1 and 13 after therapy were evaluated, so transient or increased effects affecting diffusion may have been missed.

The perfusion fraction (*f*) showed a significant correlation with the fractional tissue area of fibrosis (FD). Tissue response to irradiation may result in fibrotic tissue formation, and the GOT1 tumour model has demonstrated fibrosis in response to ^177^Lu‐ocreotate therapy, as seen on MT staining.^16^, ^37^ Few studies have correlated *f* with fibrosis in tumours. Residual lesions demonstrated significantly higher *f* in post‐chemoradiotherapy fibrosis compared with untreated tumours in nasopharyngeal carcinoma patients,^38^ which is consistent with our results, but opposite correlations have been demonstrated in liver fibrosis.^39^ The role of *f* in tumour characterization thus remains unclear, and further efforts to optimize acquisition and post‐processing methods for IVIM‐DWI are probably required before *f*, as well as D*, can be properly understood and evaluated.

The heat maps of the histological indices (Figure 3k) are informative regarding the heterogeneity of the tumour vasculature and viability. As previously mentioned, the use of CD31‐based MVD as a measure of vascular functionality and perfusion may not be optimal. MVD is mostly seen in the tumour periphery, whereas apoptosis and proliferation seem more evenly distributed throughout the tumour (Figure 3k), both of which would require vascular supply. A measure of the functional properties or maturity of the vasculature, instead of a static measure of endothelial cell presence (MVD), may better reflect perfusion.^32^, ^33^, ^34^

Some potential limitations of this study should be noted. 1) Some correlations may have been obscured by the temporal discordance between DCE‐MRI experiments (day 8) and tissue extraction (day 13) in 4/5 tumours. It is likely that the tumour biological characteristics evolved between day 8 and 13, which may have led to false negative results, *i.e*. reduced or missed correlations. However, more than 50% of the data were from tumour 2, imaged and harvested on the same day. 2) The analyses were based on data from five tumours only. This was partly due to the index calculations requiring lengthy data processing due to the immense amount of data associated with digitized histological images of microscopic resolution. Furthermore, our own tumour inclusion criteria, namely high image quality, minimal artefacts on histological sections, and adequate landmarks for proper image registration, restricted the number of tumours for evaluation. However, the variation of indices within tumours seems greater than the variation between tumours with some exceptions (Figure 4), which indicates that additional tumours would add little information. 3) As mentioned above, a lack of landmarks in central tumours reduced the amount of data from those regions, which may have limited the range of biological characteristics evaluated. To address this problem, other methods to ascertain proper registration are required. 4) No quantitative evaluation of registration accuracy was made in this study, and the landmark‐based approach for image registration entails a risk of introducing false correlations. A more objective approach would be to use non‐lesion features, such as markers outside the tumour, but intra‐tumour registration may be degraded by such an approach. 5) The 2D representation of the tumour tissue provided by the histological sections makes the method insensitive to tumour heterogeneity in the dimension of the MR slice thickness. Since biological features investigated can be seen to vary even along the dimensions of a sample (*e.g*., Figure 3), it is obvious that variations along the distance of an MR slice (4 times the sample dimensions) will influence the accuracy of the index calculations. A possible remedy would be to collect more histological sections within the distance covered by the MR slice, with enough separation to get a better representation of the imaged tumour tissue. For this to improve the accuracy, however, would require better knowledge of the precise location of the beginning and end of the acquired image slice, and thus a more sophisticated system for tracking those locations than offered by manual application of tissue ink or similar fiducial markers. There are also substantial processing times and costs involved in histological processing and post‐processing of the digitized images that would have to be considered.

## 6 CONCLUSIONS

In this work, MR parameters are correlated with spatially registered histological parameters of the imaged tumour tissue. Registration problems due to MR artefacts or morphologically distorted histological sections were minimized by sequential registration of tumour sub‐regions, and objective evaluation on a substantial amount of data samples by statistical regression methods was enabled by the supervised automatic histological indexing method.

Predictive power and statistical significance were high for several of the correlations found between MR parameters and histological indices. Models predicting HEcount and Ki67count had the strongest predictive power, while models describing MVD and FD had slightly lower predictive power. Altogether, HEcount could be predicted by three DCE‐MRI parameters related mainly to perfusion (*TOP*, *TTP,* and *BE*); Ki67count could be predicted by one DCE‐MRI parameter (*IS*, reflecting perfusion) and one relaxation parameter related to macromolecular content (*T1*); MVD could also be predicted by *T1*, and by a DCE‐MRI parameter reflecting the amount of contrast reaching the tissue (*SEmax*); and FD could be predicted by one IVIM‐DWI parameter reflecting fractional volume of actively perfused tissue (*f*). In total, seven of the evaluated MR parameters were capable of predicting apoptosis, proliferation, vessel density or fibrosis in the studied tumour type. Apart from the similarly defined *TOP* and *TTP,* the data provided by these MR parameters seem, based on cluster analysis, to contain unique information.

This work demonstrates the importance of combining data from several MR methods for gaining knowledge of tumour tissue characteristics, and we believe the information contained in this work will be valuable for further studies on mpMR evaluation of tumour tissue and response assessment. Further work is needed, such as improvements of registration procedures and MR post‐processing methods.

## FUNDING

This work was supported by the Swedish Research Council [grant number 21073]; the Swedish Cancer Society [grant number 2016/692]; BioCARE – a National Strategic Research Program at the University of Gothenburg; the King Gustav V Jubilee Clinic Cancer Research Foundation; the Sahlgrenska University Hospital Research Funds; the Assar Gabrielsson Cancer Research Foundation; the Adlerbertska Research Fund; the Wilhelm and Martina Lundgren science trust fund; the Royal Society of Arts and Sciences in Gothenburg (KVVS).

## Supporting information

Data S1. Supplemental 1. Total amount of samples (n) underlying the correlations from the mixed‐effects regression analysis, and the distribution of samples over tumours (y‐axis). Sample numbers for the tumour extracted day 1 (tumour 2) is shown in blue in the left column, separated from the other tumours, which are stacked in the right column. Note that HEcount could not be extracted from tumour 2
