# Supplementary figures and images for: Multiparametric MR for non‐invasive evaluation of tumour tissue histological characteristics after radionuclide therapy
Source: NMR Biomed. 2019 Jan 28;32(3):e4060. doi: 10.1002/nbm.4060 (PMC6590232; doi:10.1002/nbm.4060)

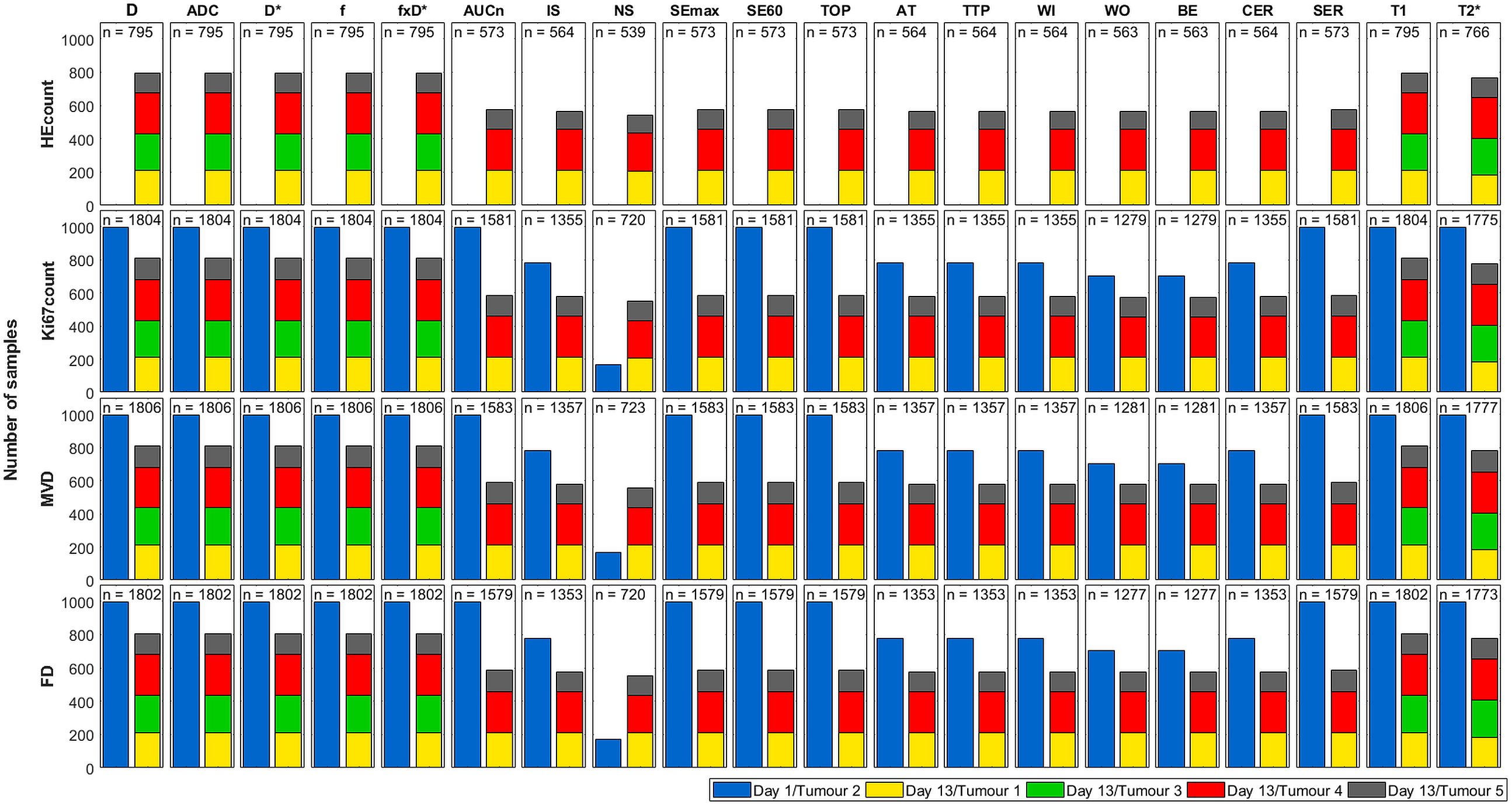

Supplement: Supplementary file 1 — Data S1. Supplemental 1. Total amount of samples (n) underlying the correlations from the mixed‐effects regression analysis, and the distribution of samples over tumours (y‐axis). Sample numbers for the tumour extracted day 1 (tumour 2) is shown in blue in the left column, separated from the other tumours, which are stacked in the right column. Note that HEcount could not be extracted from tumour 2 [file NBM-32-na-s001.tif]
